# Comparison of trend analysis of varicella zoster disease burden between China and the world 1990–2021 and disease burden forecast 2030

**DOI:** 10.3389/fpubh.2025.1535977

**Published:** 2025-03-10

**Authors:** Zhichun Chang, Huale Li, Yanfang Li, Ting Qin, Mingren Hu, Xinjing Yang, Jun Li, Yufeng Xie

**Affiliations:** ^1^Shenzhen Hospital (Futian) of Guangzhou University of Chinese Medicine, Shenzhen, China; ^2^The Sixth Clinical Medical College, Guangzhou University of Chinese Medicine, Shenzhen, China

**Keywords:** varicella zoster disease, trend, incidence, mortality, prevalence, disability-adjusted life years

## Abstract

**Objectives:**

This study aims to analyze temporal trends in the age- and sex-specific burdens of varicella zoster virus (VZV), including incidence, prevalence, mortality, and disability-adjusted life years (DALYs) in China from 1990 to 2021, and to predict the burden of varicella zoster in China for 2030 by comparing the trends with the global burden of the disease.

**Methods:**

Data from the Global Burden of Disease database (1990–2021) were used to analyze the characteristics of varicella zoster virus (VZV) burden in China and globally, including trends in incidence, prevalence, mortality, and disability-adjusted life years (DALYs). The average annual percentage change (AAPC) and its corresponding 95% confidence interval (95% CI) were calculated using Joinpoint to assess the VZV burden trends. A comprehensive comparative analysis of the differences in VZV burden between China and the global population was conducted across multiple dimensions, including age, gender, and time period. Additionally, an autoregressive integrated moving average (ARIMA) model was used to predict the VZV trend from 2021 to 2030.

**Results:**

Between 1990 and 2021, the age-standardized incidence rate (ASIR) of varicella zoster in China decreased from 1,274.93/100,000 to 1,270.58/100,000, while the global ASIR increased from 1,244.05/100,000 to 1,248.59/100,000. In China, the age-standardized prevalence rate (ASPR) decreased slightly from 72.27/100,000 to 72.03/100,000, whereas the global ASPR rose from 66.67/100,000 to 67.16/100,000. The age-standardized disability-adjusted life year rate (ASDR) in China decreased significantly, from 17.68/100,000 to 4.66/100,000, while the global ASDR decreased from 19.28/100,000 to 12.31/100,000. Similarly, China’s age-standardized mortality rate (ASMR) declined significantly, from 0.40/100,000 to 0.05/100,000, while the global ASMR decreased from 0.35/100,000 to 0.19/100,000. Over the same period, the average annual percentage change (AAPC) of ASIR, ASPR, ASMR, and ASDR in China was −0.0056, −0.0131%, −6.84%, and −4.24%, respectively, while the global AAPC for these metrics was 0.0119, 0.0183, −1.97%, and −1.42%, respectively. Additionally, age and gender had a significant impact on the burden of varicella zoster. The trends in ASIR and ASPR were notably influenced by age, while ASMR and ASDR exhibited a significant increasing trend with age. Projections indicate that the ASDR of varicella zoster in China will continue to decrease by 2030, while the ASIR, ASPR, and ASMR are expected to remain stable.

**Conclusion:**

Between 1990 and 2021, the incidence, prevalence, mortality, and DALYs of VZV in China demonstrated a declining trend, reflecting a relative reduction in the VZV burden. Women are more susceptible to VZV infection and face a higher risk of mortality than men. In contrast, the global disease burden remains higher than that in China. Projections suggest a slight decrease in the VZV burden in China by 2030. However, due to the country’s large and aging population, VZV will continue to pose a significant public health challenge.

## Introduction

Chickenpox and herpes zoster (HZ) are both caused by the VZV, with chickenpox representing the primary infection and herpes zoster occurring as a result of viral reactivation. Chickenpox typically manifests in childhood and is highly contagious, whereas herpes zoster is most commonly observed in adults and the older adult, triggered by reactivation of the virus that had previously latched onto the ganglia during a decline in immune function (1, 2).

The epidemiological characteristics of varicella and herpes zoster vary significantly across different regions, influenced by factors such as geographic disparities, vaccination strategies, and population aging. In recent years, the widespread implementation of varicella vaccination programs has led to a substantial decline in childhood varicella incidence. A recent study demonstrated that improving coverage and ensuring the timely administration of the two-dose varicella vaccine (VarV) regimen enhances immune protection in susceptible children (3). Specifically, vaccinated children exhibited a reduction in disease prevalence (4), while unvaccinated children experienced higher incidence rates (5). These findings collectively highlight the critical public health benefits of childhood vaccination against varicella. The incidence of shingles has been rising globally, particularly among the older adult. This trend may be attributed to factors such as the decline in immune function and the increase in chronic diseases among older individuals. With the ongoing issue of global aging, the incidence of shingles is likely to continue increasing (6, 7). In China, VZV remains a significant public health concern, with a more pronounced impact on individuals aged 60 and older. Therefore, the implementation of shingles vaccination strategies should be considered (8).

Studies suggest that older adults are generally less willing to receive vaccinations, largely due to limited knowledge about infections and vaccines, as well as negative attitudes toward vaccination. Therefore, in China, improving health education on shingles, promoting immunization, and enhancing vaccine affordability would be highly beneficial (9). A study by Diana Martins and colleagues found that the introduction of a shingles vaccination program was associated with a reduction in disease burden and a decrease in the use of related emergency medical services (10).

The studies above suggest that increasing vaccination rates among the older adult can help reduce the disease burden of shingles. This burden is reflected in its significant health impact and economic costs, including acute pain and chronic complications such as postherpetic neuralgia (PHN) (11). PHN is more common among older adult patients and significantly affects their quality of life and the utilization of social resources. Various varicella-related complications can occur frequently, impacting quality of life, healthcare resource utilization, and budgets (12). Research has shown that VZV-related neurological complications are heterogeneous, often resulting in severe disability and poor prognosis (13).

Additionally, the incidence of shingles is significantly higher in immunocompromised populations, further exacerbating the public health challenge (14). Patients with malignancies, those undergoing immunosuppressive therapy following organ transplantation, and individuals on long-term immunosuppressive medications are at an elevated risk. Due to impaired immune function in these populations, their ability to defend against varicella-zoster virus is reduced, leading to a significantly higher incidence of shingles (15). Other studies have found that individuals with compromised immune function are also at increased risk of developing neurological complications, such as neuralgia (14).

This study aims to analyze the incidence, prevalence, mortality, and DALYs of varicella and shingles in China and globally from 1990 to 2021 by using data from the Global Burden of Disease (GBD) database, and to compare long-term trends between China and the world. By examining the influence of socioeconomic development, vaccination strategies, and population aging on disease burden, this study will provide scientific evidence to inform the development of targeted policies and the optimization of resource allocation.

## Methods

### Data source

The data for this study were sourced from the 2021 Global Burden of Disease (GBD) database. Developed by the Institute for Health Metrics and Evaluation (IHME), the database includes indicators for a wide range of diseases and injuries, offering multidimensional, cross-temporal data on the global burden of disease, which are suitable for assessing incidence, prevalence, mortality, and DALYs (16). We retrieved data on VZV from the GBD database for the period 1990–2021, covering both China and global data. First, we selected the categories “Cause of Death or Injury,” “Varicella,” and “Herpes Zoster” from the GBD 2021 database on the official website. We then specified the relevant age groups, regions, and time intervals before downloading the data. Since this study utilizes publicly available data, ethical approval or informed consent was not required.

### Statistical analysis

In this study, we extracted data on the incidence, prevalence, mortality, DALYs, and corresponding age-standardized rates of VZV in China and globally from the GBD database. For statistical analysis, we utilized Joinpoint software (version 5.1.0) to calculate the average annual percentage change (AAPC) and its 95% confidence intervals (95% CI) to assess the temporal trends in the VZV burden (17, 18). The age-standardized indices were logarithmically fitted using the regression model ln (y) = *α* + *β*x + *ε*, where y represents the corresponding age-standardized index, x represents the calendar year, and the average annual percentage change (AAPC) is calculated as 100 × (exp (β) − 1), with its 95% confidence intervals (CIs) also derived from the model. If the 95% CI of the AAPC is greater than 0, the age-standardized indicator indicates an increasing trend; if less than 0, it suggests a decreasing trend; and if it includes 0, it implies a stable trend. Predictive analyses were conducted using autoregressive integrated moving average (ARIMA) models to forecast VZV trends from 2021 to 2030. The ARIMA model is a time series method where, in the ARIMA (p, d, q) model, “p” represents the number of autoregressive terms, “d” denotes the degree of differencing, and “q” represents the number of moving average terms. Initially, differencing was applied to achieve stationarity in the time series data, and the Kwiatkowski-Phillips-Schmidt-Shin (KPSS) test was used to confirm stationarity. The Bayesian Information Criterion (BIC) was then used to compare the goodness-of-fit of different models. Finally, the Ljung-Box test assessed the robustness of the residuals. The model was considered optimal for short-term time series forecasting when the residuals exhibited randomness (19). Statistical analysis and data visualization were performed using R statistical software (version 4.4.1) and Joinpoint software. A *p*-value of less than 0.05 was considered statistically significant in this study.

## Results

### Description of China and global VZV burden

#### Incidence and prevalence of VZV in China and worldwide

From 1990 to 2021, the population-wide incidence of varicella zoster in China showed a slight decrease, with the number of cases dropping from 13.88 million (95% CI: 13.18–14.62 million) to 12.5 million (95% CI: 11.11–13.90 million). The ASIR decreased slightly from 1,274.93/100,000 to 1,270.58/100,000 (AAPC = −0.0056, 95% CI: −0.0294 to 0.0182%), reflecting a relatively stable trend. The number of prevalent cases increased from 755,000 (95% CI: 636,000–894,000) to 1.12 million (95% CI: 906,000–1.36 million), while the ASPR decreased slightly from 72.27/100,000 to 72.03/100,000 (AAPC = −0.0131, 95% CI: −0.0321 to 0.0058%). Globally, the population-wide incidence of varicella zoster increased from 72.83 million (95% CI: 70.11–75.85 million) to 86.67 million (95% CI: 81.68–92.21 million), and the ASIR increased from 1,244.05/100,000 to 1,248.59/100,000 (AAPC = 0.0119, 95% CI: 0.0058 to 0.016%). The number of prevalent cases rose from 3.37 million (95% CI: 2.92–3.89 million) to 5.28 million (95% CI: 4.41–6.18 million), with a slight increase in the ASPR from 66.67/100,000 to 67.16/100,000 (AAPC = 0.0183, 95% CI: −0.0039 to 0.0406%) (Table 1).

**Table 1:** All-age cases and age-standardized incidence, prevalence, mortality and DALYs rates for VZV in China and globally in 1990 and 2021, and corresponding AAPCs.

Location	Measure	1990		2021		1990–2021 AAPC
		All-ages cases	Age-standardized rates per 100,000 people	All-ages cases	Age-standardized rates per 100,000 people	
		*n* (95% CI)	*n* (95% CI)	*n* (95% CI)	*n* (95% CI)	*n* (95% CI)
China	Incidence	13,887,584	1,274.929	12,504,897	1,270.58	−0.0056
		(13,183,440–14,620,874)	(1,211.66–1,344.98)	(11,110,002–13,989,387)	(1,208.53–1,339.47)	(−0.0294–0.0182)
	Prevalence	754,742	72.266	1,116,219	72.029	−0.0131
		(635,860–893,851)	(61.166–85.11)	(905,617–1,360,149)	(60.92–84.925)	(−0.0321–0.0058)
	DALYs	176,922	17.681	71,507	4.661	−4.2445
		(152,341–204,286)	(15.296–20.23)	(48,331–100,573)	(3.324–6.301)	(−4.413–4.0754)
	Deaths	2,666	0.402	644	0.046	−6.8396
		(2,387–2,951)	(0.351–0.451)	(537–755)	(0.038–0.053)	(−7.0088–6.6701)
Global	Incidence	72,830,736	1,244.051	86,678,087	1,248.592	0.0119
		(70,112,715–75,848,873)	(1,187.62–1,303.325)	(81,687,121–92,207,569)	(1,192.39–1,309.92)	(0.008–0.016)
	Prevalence	3,371,208	66.667	5,282,097	67.161	0.0183
		(2,914,534–3,885,486)	(56.809–77.224)	(4,407,183–6,182,740)	(56.986–77.814)	(−0.0039–0.0406)
	DALYs	1,065,063	19.284	886,067	12.309	−1.4224
		(936,651–1,230,333)	(17.01–22.054)	(744,313–1,060,072)	(10.362–14.734)	(−1.4943–1.3504)
	Deaths	15,633	0.354	13,931	0.191	−1.9661
		(14,141–17,385)	(0.321–0.384)	(12,585–15,605)	(0.171–0.215)	(−2.0277–1.9045)

### Deaths and DALYs of VZV in China and globally

In China, the number of deaths due to varicella zoster declined significantly from 2,666 (95% CI: 2,387–2,951) in 1,990 to 644 (95% CI: 537–755) in 2021. The ASMR decreased from 0.402/100,000 to 0.046/100,000 (AAPC = −6.8396, 95% CI: −7.0088% to −6.6701%). The corresponding number of DALYs decreased from 177,000 (95% CI: 152,000–204,000) to 72,000 (95% CI: 48,000–101,000), with the ASDR decreasing from 17.68/100,000 to 4.66/100,000 (AAPC = −4.2445, 95% CI: −4.413% to −4.0754%). Globally, the number of deaths from varicella zoster decreased from 15,600 (95% CI: 14,100–17,400) to 13,900 (95% CI: 12,500–15,600), while the ASMR decreased from 0.354/100,000 to 0.191/100,000 (AAPC = −1.9661, 95% CI: −2.0277% to −1.9045%). Concurrently, the global number of DALYs decreased from 1.065 million (95% CI: 937,000–1.230 million) to 886,000 (95% CI: 744,000–1.060 million), and the ASDR decreased from 19.28/100,000 to 12.31/100,000 (AAPC = −1.4224, 95% CI: −1.4943% to −1.3504%) (Table 1).

### Joinpoint regression analysis of VZV burden in China and globally

**Figure 1:**
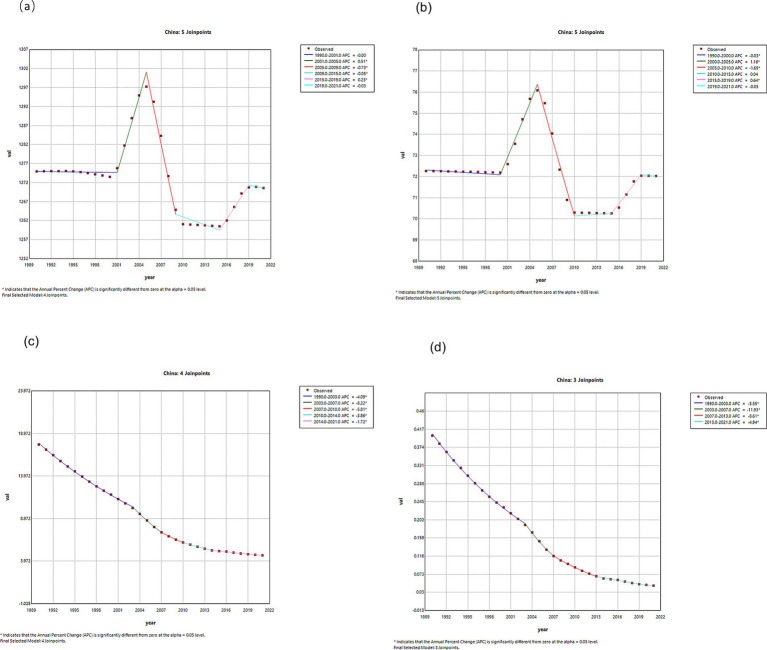
APCs of ASIR, ASPR, ASDR and ASMR of VZVs in China from 1990 to 2021 (*indicates *p*-value <0.05). **(a)** ASIR; **(b)** ASPR; **(c)** ASDR; **(d)** ASMR.

**Figure 2:**
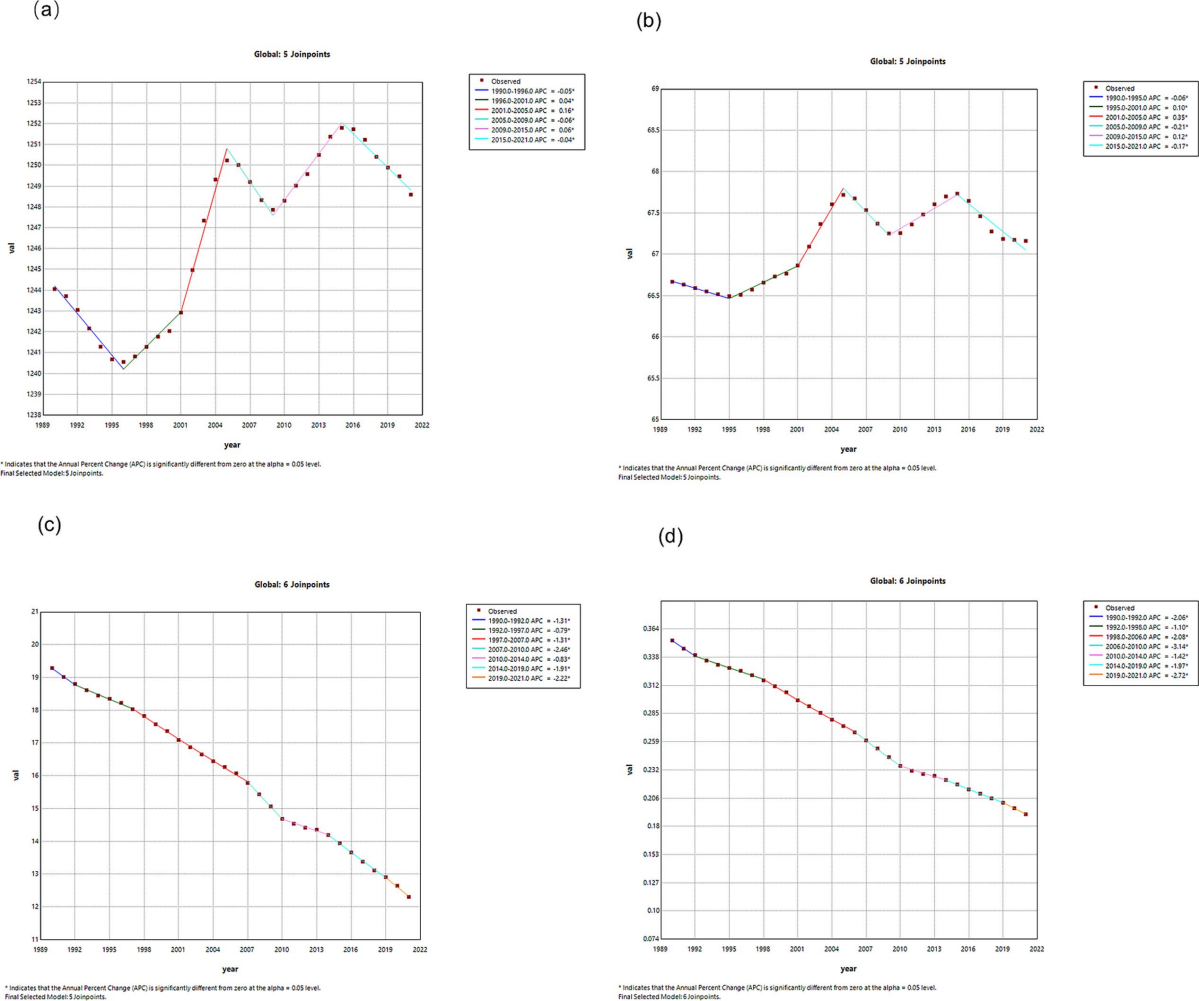
APCs of ASIR, ASPR, ASDR and ASMR for global VZVs from 1990 to 2021 (*indicates *p*-value <0.05). **(a)** ASIR; **(b)** ASPR; **(c)** ASDR; **(d)** ASMR.

Joinpoint regression analyses of the ASIR, ASPR, DALYs, and ASMR for VZV in China and globally are shown in Figures 1, 2. In China, the incidence and prevalence of VZV remained stable from 1990 to 2001, then increased from 2000 to 2005, with annual percentage changes (APCs) of 0.15 and 1.16 (*p* < 0.05). From 2005 to 2009, a decreasing trend was observed (*p* < 0.05). The trend reversed from 2015 to 2019, showing an increase (*p* < 0.05), and stabilized in 2019. Mortality and DALYs have been on a downward trend from 1990 to 2021. Globally, the incidence and prevalence decreased from 1990 to 1995, then increased from 1996 to 2005, followed by a decrease from 2005 to 2009, with APCs of −0.06 and −0.21 (*p* < 0.05), respectively. From 2009 to 2015, both incidence and prevalence increased, with APCs of 0.06 and 0.12 (*p* < 0.05), respectively. From 2015 to 2021, a decreasing trend was observed, with APCs of −0.04 and 0.17 (*p* < 0.05), respectively.

### Trends in VZV burden in China and globally

**Figure 3:**
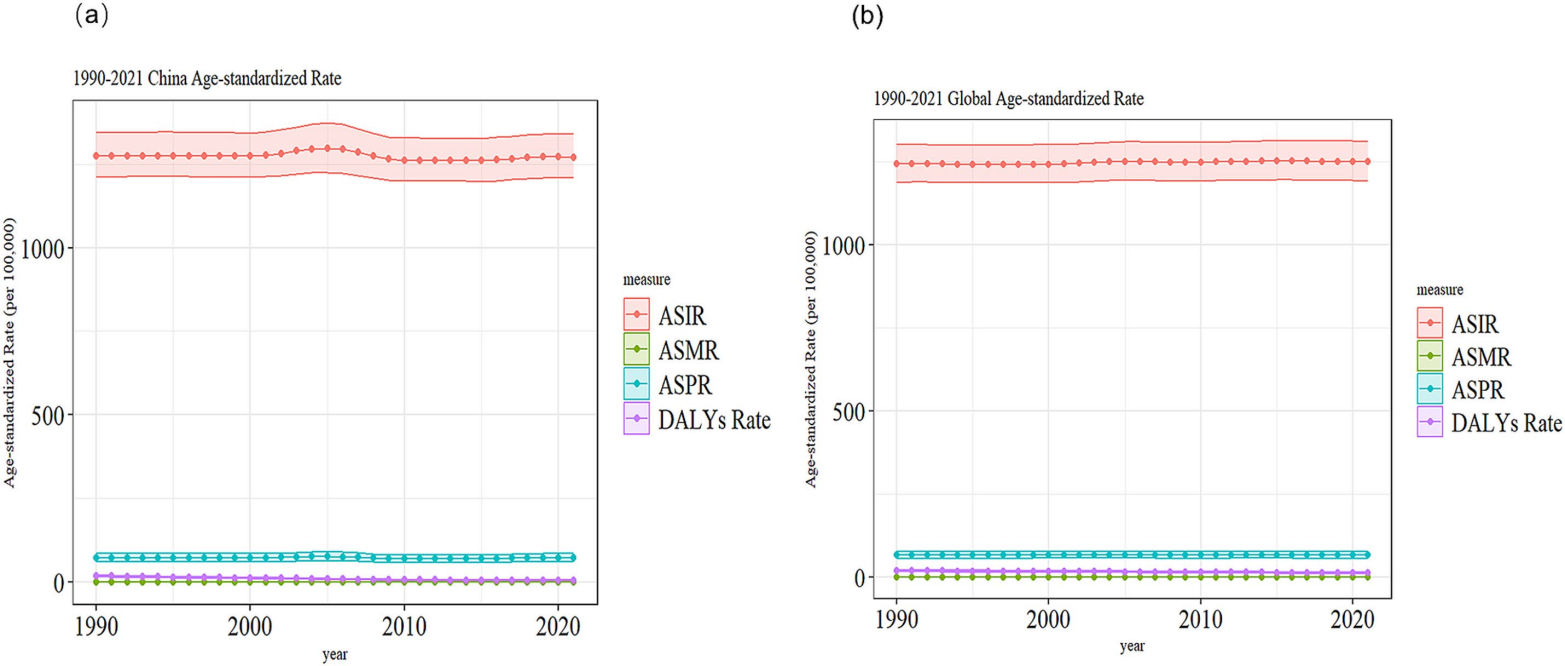
Comparison of the trends of ASIR, ASPR, ASDR and ASMR of VZVs in China and globally from 1990 to 2021. **(a)** China; **(b)** Global.

In Figure 3a, the ASIR and ASPR in China show a slight increase from 1990 to 2005, followed by a decrease, while the ASMR and ASDR exhibit a gradual decline from 1990 to 2021. In Figure 3b, both ASDR and ASMR show a small decrease from 1990 to 2021, while ASIR and ASPR display negligible changes.

### Disease burden of VZV in different age groups in China, 1990 and 2021

**Figure 4:**
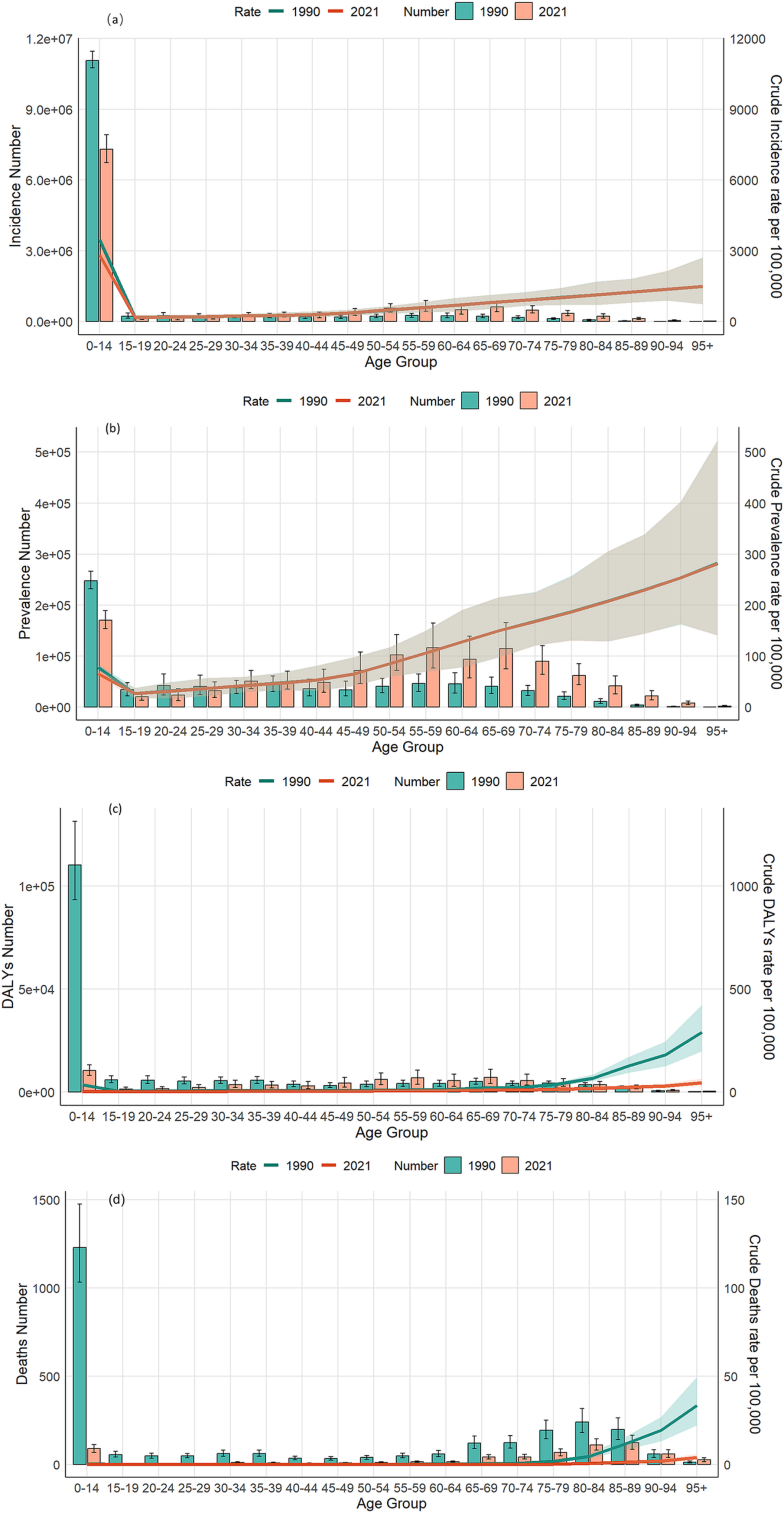
Comparison of incidence, prevalence, number of deaths, DALYs and their crude rates by age group in China from 1990 to 2021. **(a)** Number of incidences and CIR; **(b)** number of prevalence and CPR; **(c)** number of DALYs and CDR; **(d)** number of deaths and CMR.

The incidence, prevalence, DALYs, mortality rates, and corresponding crude rates of VZV across all age groups in China from 1990 to 2021 are shown in Figure 4. In Figure 4a, the highest prevalence and number of cases are observed in the 0–14 years age group, with both prevalence and case numbers significantly lower in 2021 compared to 1990. Prevalence gradually increases with age after 14 years. In Figure 4b, the highest prevalence and number of cases are also seen in the 0–14 years group. The main age group for incidence is concentrated in the 50–80 years range, while the crude incidence rate shows an upward trend. Figure 4d illustrates that the number of deaths is concentrated in the 75–89 years age group, with both the number of deaths and the mortality rate in 2021 significantly lower than in 1990.

### Gender differences in the burden of VZV disease in different age groups in China

**Figure 5:**
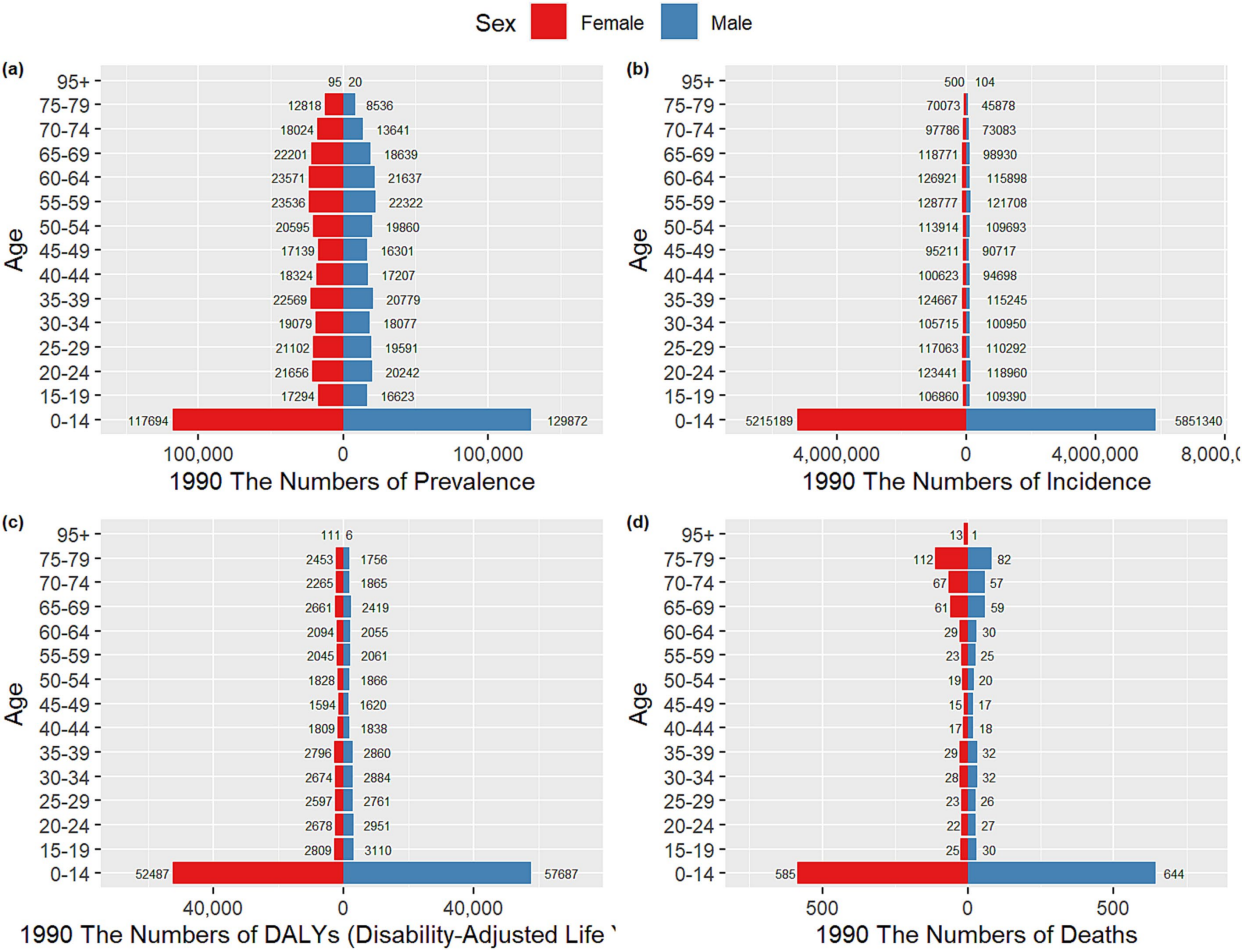
Comparison of VZV incidence, prevalence, mortality, and DALYs for males and females in different age groups in China in 1990. **(a)** Incidence; **(b)** prevalence; **(c)** DALYs; **(d)** mortality.

**Figure 6:**
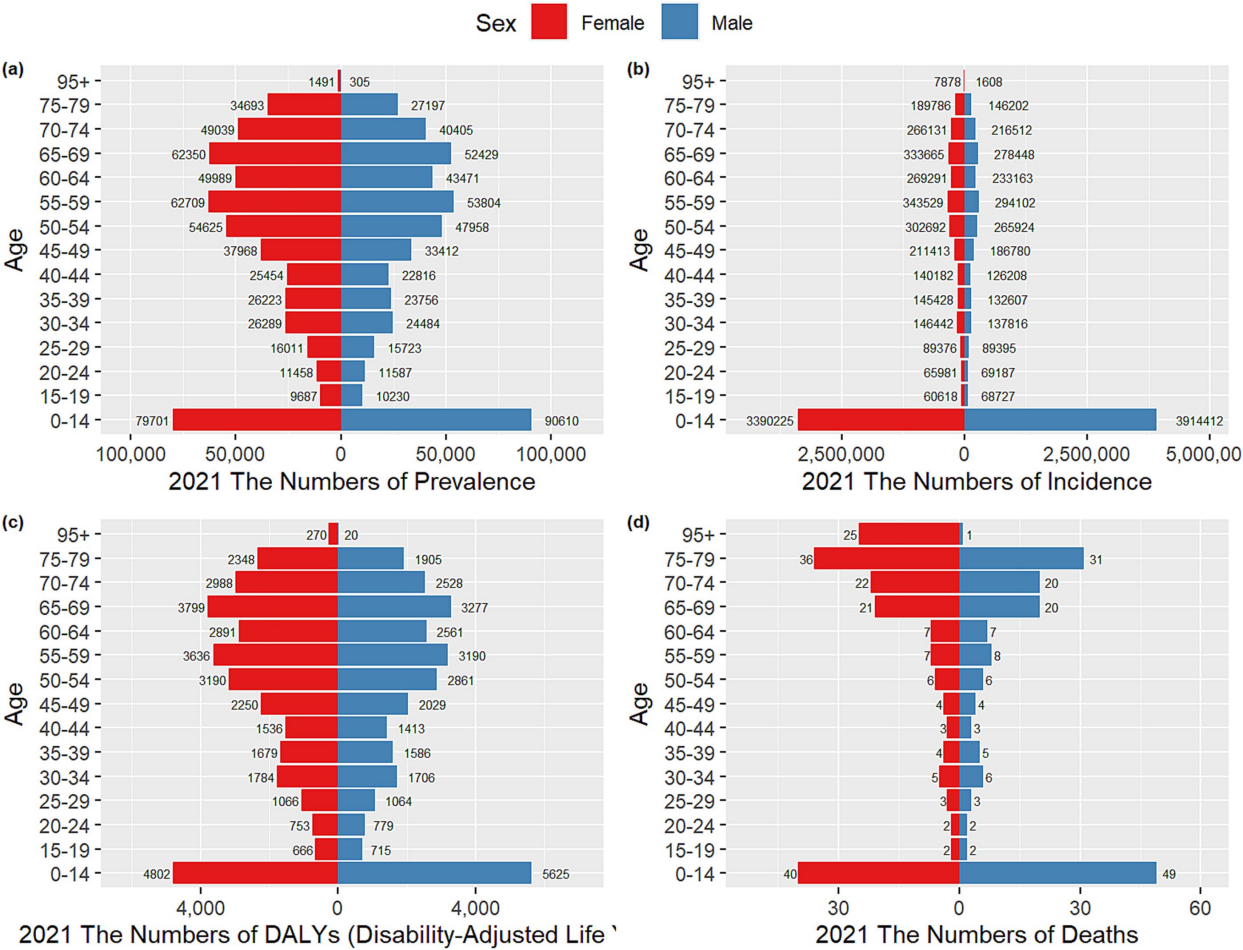
Comparison of VZV incidence, prevalence, mortality and DALYs for men and women in different age groups in China in 2021. **(a)** Incidence; **(b)** prevalence; **(c)** DALYs; **(d)** mortality.

**Figure 7:**
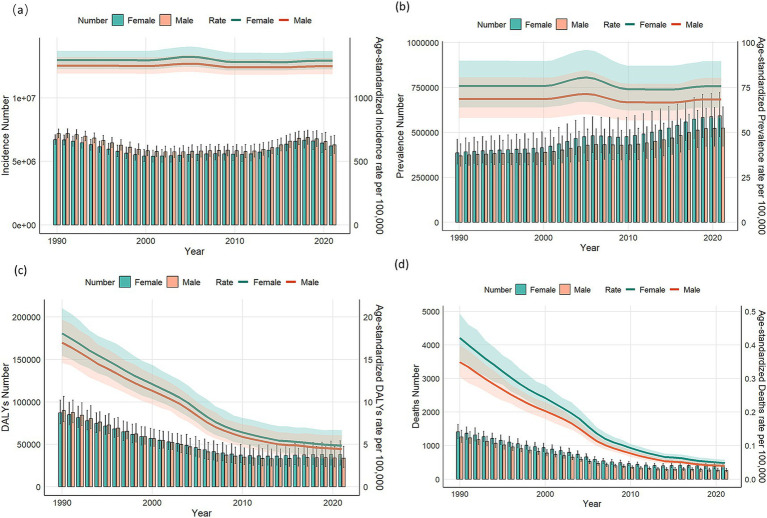
Comparison of age-standardized incidence, prevalence, mortality, and disability-adjusted life years (DALYs) for all-age cases for males and females in China from 1990 to 2021. **(a)** Incidence cases and ASIR; **(b)** prevalence cases and ASPR; **(c)** DALYs counts and ASDR; **(d)** deaths and ASMR. Bar graph representation bars represent counts; lines represent age-standardized rates.

Figures 5, 6 present the prevalence, morbidity, mortality, and DALYs of VZV for males and females across different age groups in China in 1990 and 2021. From Figure 5, it is evident that the highest prevalence, morbidity, mortality, and DALYs are concentrated in the 0–14 years age group, with males outnumbering females in all measures. The number of cases increases with age, peaking at the 55–59 years age group. Figure 6 shows that all data improved in 2021 compared to 1990, with a consistent overall trend. Figure 7 compares the burden of disease and age-standardized incidence of VZV among men and women of all ages in China from 1990 to 2021. The incidence and prevalence showed an upward trend from 2000 to 2005, followed by a gradual decline after 2005, and stabilization after 2010. The prevalence has exhibited a slight increase from 1990 to 2021. Meanwhile, the mortality rate, number of deaths, DALY rate, and total DALYs have all shown a significant downward trend year by year from 1990 to 2021.

### Projected disease burden in China over the next 10 years

**Figure 8:**
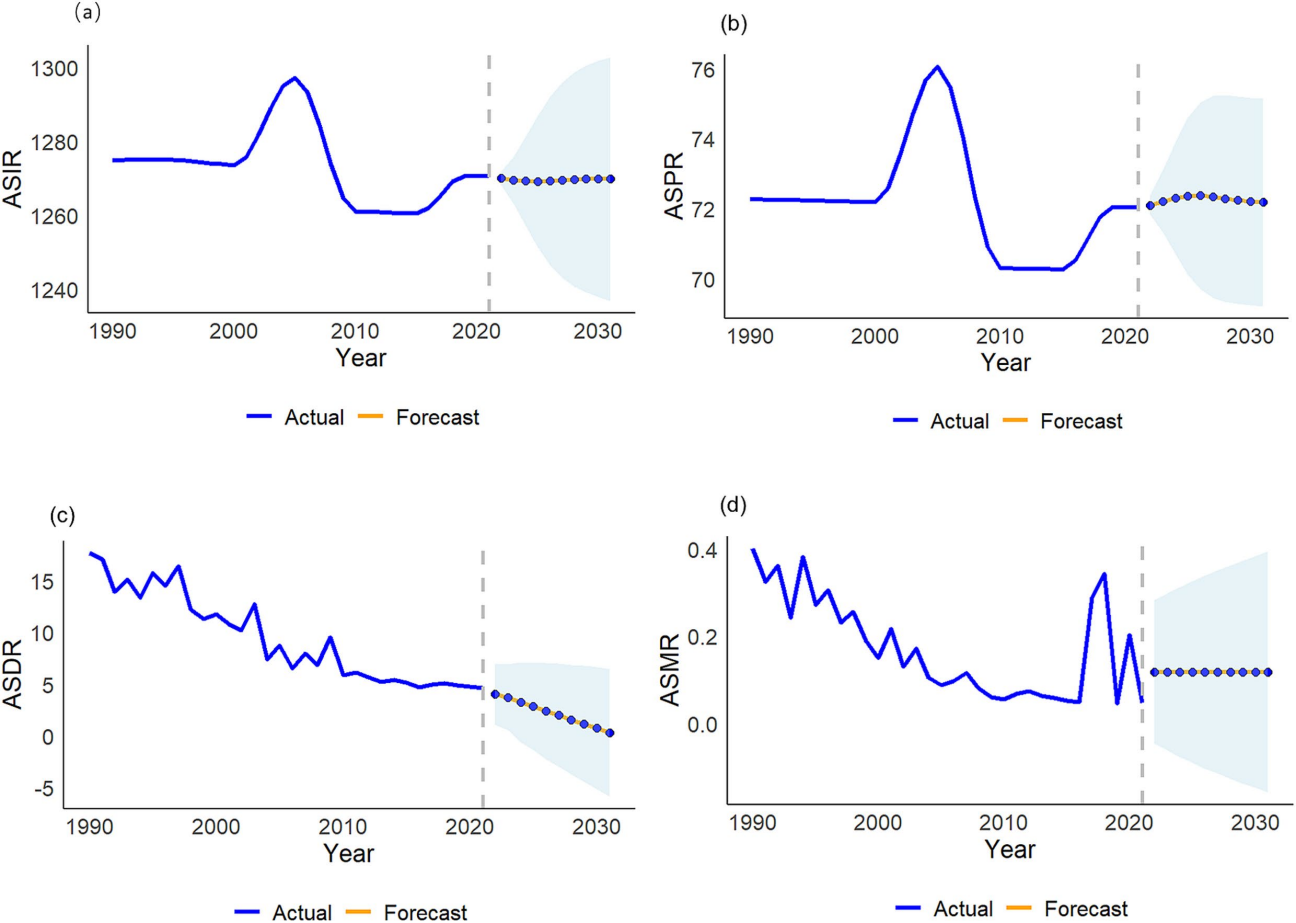
Projected burden of disease in China over the next 10 years. **(a)** ASIR; **(b)** ASPR; **(c)** ASDR; **(d)** ASMR.

From Figure 8, it can be seen that by 2030 the ASDR of VZV diseases in China will further decrease, the ASPR will slightly decrease, while the ASIR and ASMR will remain stable.

## Discussion

This study provides a comprehensive analysis of the VZV disease in China and globally from 1990 to 2021, revealing significant temporal trends as well as age, gender, and regional differences in incidence, prevalence, mortality, and DALYs. Notably, in China, while the ASIR and ASPR have remained relatively stable, both the ASMR and ASDR have significantly declined. This trend contrasts with the global experience and warrants particular attention. Globally, the ASIR and ASPR have increased, while reductions in mortality and DALYs have been relatively modest. These divergent trends highlight the success of public health interventions in China, while also emphasizing the continued global challenge posed by VZV (6). This disparity is likely linked to several factors, including vaccination policies, healthcare accessibility, population aging, and variations in public health strategies.

China has made significant progress in expanding varicella vaccination coverage among children. Studies have shown that the introduction of the varicella booster vaccine led to a marked increase in VZV antibody levels among children in Beijing (20). In Shanghai, a two-dose varicella vaccine (VarV) immunization program has been widely implemented, with free vaccination becoming available in August 2018. Following the introduction of this program, there was a notable reduction in the number of varicella cases among children (21). The varicella vaccine has increasingly been incorporated into provincial immunization programs, leading to a decline in primary varicella cases and, as a result, reducing the future risk of herpes zoster reactivation in these populations.

Globally, vaccination coverage and policies vary significantly. In high-income countries such as the United States, Canada, and several European nations, vaccination rates for varicella and herpes zoster are relatively high, leading to a lower disease burden in these regions (4). In contrast, many low- and middle-income countries face low vaccination coverage due to factors such as high vaccine costs, unstable supply chains, and limited public awareness, resulting in persistently high incidence and mortality rates of herpes zoster (5, 6).

In recent years, the Chinese government has increased its investment in the healthcare sector, particularly in the development of the primary healthcare system. This has enhanced the accessibility of medical services, enabling more individuals to receive timely diagnoses and treatment. Furthermore, China has actively promoted the equalization of public health services, ensuring that residents in remote and rural areas also have access to essential healthcare services (22, 23). Globally, the distribution of healthcare resources remains highly unequal. Developed countries benefit from advanced medical facilities and a well-equipped workforce, whereas many developing nations face critical shortages of healthcare resources. This disparity has led to significant differences in the effectiveness of disease prevention and control efforts (24, 25).

China is experiencing rapid population aging, leading to a growing older adult population that is more susceptible to herpes zoster. This study observed a slight increase in the number of cases in China. While the country has mitigated some of the adverse effects of aging through improved healthcare management for the older adult, the accelerating aging process presents a significant challenge for the nation in the future (26). Globally, aging is a widespread trend, and the decline in immune function among the older adult places them at high risk for herpes zoster, which often manifests more severely and is accompanied by additional complications. As a result, aging has become a key driver of the increasing disease burden of herpes zoster worldwide (27).

Age subgroup analyses clearly demonstrate the biphasic nature of the VZV disease burden. Understanding the dual-phase progression of VZV infection is essential, as primary infection (varicella) typically occurs in childhood, when the immune system is still developing and resistance to VZV is relatively weak (28). The virus then enters a latent phase, with reactivation commonly occurring as herpes zoster in older adults or individuals with compromised immune function (29). This fundamental characteristic of VZV’s natural history underpins the age-related patterns observed in the disease burden. As individuals age, there is a notable increase in disease burden among middle-aged and older adult populations, underscoring the need for age-specific interventions (13, 14). Studies have shown that the incidence and hospitalization rates of herpes zoster rise with age, particularly after 50 (15, 30).

Gender distribution data indicate that the ASIR and ASMR are typically higher in females than in males. This may reflect the influence of gender on disease prevalence, consistent with previous studies suggesting that female sex is an independent risk factor for herpes zoster (31). A community-based prospective cohort study found that herpes zoster incidence was higher in females than in males, potentially due to differences in immune metabolism and hormonal levels (32).

In conclusion, this study demonstrates that China has made significant progress in controlling the VZV disease burden, particularly through reductions in DALYs and mortality rates. However, with population aging and a shifting disease burden toward the adult population, new challenges are emerging. Globally, despite a general decline in disease burden, the rising number of cases and regional disparities in vaccination coverage remain significant concerns. Moving forward, efforts should prioritize promoting widespread vaccination, improving access to herpes zoster vaccines and treatments, enhancing disease prevention and control, and advancing research focused on children, the older adult, and female populations. Achieving comprehensive disease control will require multi-sectoral collaboration.

## Data Availability

The original contributions presented in the study are included in the article/Supplementary material, further inquiries can be directed to the corresponding author.
